# Application of a Bio-Economic Model to Demonstrate the Importance of Health Traits in Herd Management of Lithuanian Dairy Breeds

**DOI:** 10.3390/ani12151926

**Published:** 2022-07-28

**Authors:** Šarūnė Marašinskienė, Rūta Šveistienė, Barbara Kosińska-Selbi, Christin Schmidtmann, Jehan Frans Ettema, Violeta Juškienė, Morten Kargo

**Affiliations:** 1 Animal Science Institute, Lithuanian University of Health Sciences, R. Žebenkos 12, LT-82317 Baisogala, Lithuania; ruta.sveistiene@lsmuni.lt (R.Š.); violeta.juskiene@lsmuni.lt (V.J.); 2 Biostatistics Group, Department of Genetics, Wrocław University of Environmental and Life Sciences, Kożuchowska 7, 51-631 Wrocław, Poland; barbara.kosinska@upwr.edu.pl; 3 Institute of Animal Breeding and Husbandry, Kiel University, Hermann-Rodewald-Str. 6, 24118 Kiel, Germany; cschmidtmann@tierzucht.uni-kiel.de; 4 SimHerd A/S, Niels Pedersens Alle 2, DK-8830 Tjele, Denmark; je@simherd.com; 5 Center for Quantitative Genetics and Genomics, Aarhus University, Blichers Allé 20, DK-8830 Tjele, Denmark; morten.kargo@qgg.au.dk

**Keywords:** bio-economic model, breeding goal, dairy cattle, economic value, herd health management

## Abstract

**Simple Summary:**

The aims of dairy cattle breeding are more often associated with direct health evidence in relation to the net financial gain and the weighting factors are usually economic values that are retrieved from a model of a dairy herd production system. In our study we used a stochastic bio-economic model SimHerd, which allows us to derive economic values for production, fertility, calving, the survival of cows and calves and assign the importance of health traits to the economic values. Special emphasis was placed on the economics values of health traits and their importance for Lithuanian dairy cattle.

**Abstract:**

Assessing the economic importance of traits is crucial for delivering appropriate breeding goals in dairy cattle breeding. The aim of the present study was to calculate economic values (EV) and assign the importance of health traits for three dairy cattle breeds: Lithuanian Black-and-White open population (LBW), Lithuanian Red open population (LR) and Lithuanian Red old genotype (LROG). The EV estimation was carried out using a stochastic bio-economic model SimHerd, which allows the simulation of the expected monetary gain of dairy herds. The simulation model was calibrated for LBW, LR and LROG breeds, taking into account breed-specific phenotypic and economic data. For each trait, two scenarios were simulated with a respective trait at different phenotypic levels. To obtain the EVs, the scenarios were compared with each other in terms of their economic outcomes. In order to avoid the double counting of the effects, the output results were corrected using a multiple regression analysis with mediator variables. The EVs were derived for the traits related to production ECM (energy-corrected milk), fertility, calving traits, calf survival, cow survival and direct health. To demonstrate the importance of health traits in herd management, we provided reliable EVs estimates for functional traits related to herd health. The highest EV for direct health traits, caused by an increase in of 1 percentage point, were those found for mastitis (EUR 1.73 to EUR 1.82 per cow-year) and lameness (EUR 1.07 to EUR 1.27 per cow-year). The total costs per case of ketosis, milk fever and metritis ranged from EUR 1.01 to EUR 1.30, EUR 1.14 to EUR 1.26 and EUR 0.95 to EUR 1.0, respectively. The highest economic values of dystocia were estimated for LROG (EUR −1.32), slightly lower for LBW (EUR −1.31) and LR (EUR −1.23). The results of this study show the importance of health traits to the economic features of cattle herd selection of new breeding goal and this would improve the herd health. The economic evaluation of the functional traits analyzed in this study indicated the significant economic importance of the functional traits in Lithuanian dairy cattle breeds.

## 1. Introduction

In Lithuania, two main milk-type cattle breeds have been raised—Lithuanian-Black- and-White (LBW) and Lithuanian Red (LR) cows. Based on the Annual Report of Milk Recording (2021) [1], Lithuanian Red cows make up 26%, Lithuanian Black-and-White 71%, and Lithuanian Red old genotype cattle 0.04% (50 cows) of the total number of dairy cows in Lithuania. Lithuanian Black-and-White open population cattle, the largest population in Lithuania, was developed by crossbreeding local livestock with different imported breeds, such as Dutch Black-and-White, Ostfriesian and Swedish Black-and-White. LR was formed by crossing local Red cattle with different breeds, such as the Danish Red, Angler and Swedish Red-and-White, and there were also crossings with the Brown Swiss, Latvian Brown and Simmental cattle. In 1951, LR and LBW were recognized as independent breeds [2,3], but both were continuously improved by crossbreeding with international breeds. Currently, both dairy cattle breeds have become modern open populations. According to a study conducted in Lithuania, the population of Lithuanian Black-and-White cattle is dominated by 50–87.5% Holstein blood cows [4]. In order not to lose the specific genes because of intensive crossbreeding, the protection of old genotype LR and LBW cattle was started in 2001 [2].

Modern dairy cattle breeding has successfully increased production levels, but the upward trend in milk production per cow has been associated with undesirable side-effects: an increase in production diseases and reproductive problems [5]. According to Winding et al. [6], management and genetic effects are considered separately; however, genetic parameters, such as genetic correlations between production and health, may change depending on environment. In order to avoid this deterioration of functional traits, a balanced improvement of production and functional traits is required [7].

Usually, the estimated breeding values of different traits are combined into a total merit index [8,9]. A total merit index includes milk yield and several functional traits, such as calving ease, fertility, disease and longevity [7]. The term functional traits describe a set of characteristics of animals whose effect on the economic efficiency of cows is through a reduction in costs rather than an increase in product output. Functional traits, such as reproduction, longevity and health traits, were of increased interest to producers to improve herd profitability.

Currently, the aims of dairy cattle breeding are more often associated with direct health evidence in relation to the net financial gain [4,10]. In dairy cattle breeding, the weighting factors are usually economic values (EV) that are retrieved from a model of a dairy herd production system [11]. According to Wolfová and Wolf [12], an accurate definition of the regarded traits is important when calculating the EV of a trait, and the relationships between the trait of interest and other traits need to be considered and properly accounted for. Another aspect is a double counting problem, when the EV is derived using models where correlations between the traits are included. Østergaard et al. [11] proposed a solution, which helps to correct the calculated EVs of breeding traits using multiple regression analysis with mediator variables. This allows to eliminate the economic effects caused by the correlations of the simulated traits and, thus, to avoid double counting. Schmidtmann et al. [13] defined EVs as the marginal utility of a trait reflecting the direction of each trait in the breeding goals of dairy cattle and the impact on monetary profit while keeping all other traits constant.

The role of herd management and herd health is becoming increasingly important and has to meet the challenges of balancing high yield with reproductive performance and rearing healthy animals during their entire life. For instance, ketosis is a common disease in high producing dairy cows during the early lactation period with considerable associated economic costs [14]. According to Juozaitiene et al. [15], the analysis of the calving ease score in LBW dairy cows showed that 34.71% of animals needed assistance calving, of which 3.11% of cows was evaluated as needing “considerable force” or having an “extremely difficult birth”. Enting et al. [16] found that the average economic losses on the farms from clinical digital diseases per foot-lame cow (NLG 50 per average cow) averaged 21% of incidence per year in Duch dairy farms. Reduced dairy herd profitability is associated with health and fertility costs, which are also the leading causes of involuntary culling.

In Lithuania, direct health traits have not yet been officially included in the national genetic evaluation system, because direct health traits are not routinely recorded as production and calving traits. The selection index of dairy cattle consists of the following groups: productivity, exterior, somatic cell score, fertility and longevity [17], but recently, more and more attention has been paid to improve functional characteristics, such as health traits in dairy cattle breeding.

Therefore, the objective of this study was to derive EVs for production, fertility, calving, and survival of cows and calves and assign the importance of health traits to the EVs for three Lithuanian cattle breeds. In the study, special emphasis was placed on the economics values of health traits and their importance for Lithuanian dairy cattle. For this purpose, a stochastic bio-economic model SimHerd was used. SimHerd simulates the expected monetary gain in dairy cattle herds for a long period time and is thus a good basis for designing breeding goals.

## 2. Materials and Methods

The stochastic bio-economic model SimHerd [18,19,20], which simulates the expected monetary gain in dairy herds, was used. The details of the model are described in Østergaard et al. [18] and, therefore, this paper presents only a brief outline of the model. The simulation model was calibrated for the three breeds (LWB, LR and LROG), taking into account a breed-specific phenotypic and economic data from the Annual Report of Milk Recording of Lithuanian breeds. For each trait, two scenarios were simulated with the respective trait at different phenotypic levels: the current performance level of the trait parameters as recorded in the herd was increased by 1 percentage point (“high“ scenario) and decreased by 1 percentage point (“low“ scenario). To obtain the EVs, the two different scenarios were compared with each other at two levels in terms of their economic outcomes. To avoid double-counting of effects, the economic outcome was corrected using multiple regression analysis with mediator variables [11].

### 2.1. SimHerd–A Bio-Economic Model

The SimHerd model [18] is widely used in many dairy cattle modeling studies, i.e., for deriving economic values for setting breeding goals [13,21] or for investigating the economic consequences of crossbreeding [22]. The SimHerd program models milk yield, feed intake, reproduction and diseases. The simulation was performed in weekly steps. A schematic overview of the stages of the SimHerd model is given in Table 1.

### 2.2. Description of Traits

EVs were derived for 15 traits grouped in different trait categories:

1. Production. ECM (kg) was calculated on the basis of milk, fat and protein yield for LBW, LR and LROG. The study assumed that the lower use of concentrates for the LROG was due to lower yield.
2. Health traits. EVs were estimated for the following diseases: mastitis related with udder health, lameness in relation to the health of claws and legs, ketosis and milk fever associated with metabolic health and metritis representing a reproductive disorder in dairy cattle.
3. Reproduction traits. EVs were estimated for conception rate and insemination rate of both cows and heifers. Conception rate was the probability of a cow/heifer to become pregnant after insemination. The insemination rate of a cow/heifer was defined as the probability of a female to become pregnant after insemination.
4. Calving traits. Calving traits were represented by dystocia and stillbirth. Dystocia was defined as the probability of a difficult calving with veterinary assistance. Stillbirth was defined as the proportion of dead calves within 48 h postpartum as an average of both primiparous and multiparous cows.
5. Calf and cow survival traits. Calf survival (early and late) traits were represented by the probability of a calf dying in the period from 3 d to 14 d postpartum and 189 to 458 d postpartum, respectively. Cow mortality was represented as the probability of a cow dying due to a process not influenced by the health of the fertility problem.

### 2.3. Double Counting and Multiple Regression

According to Østergaard et al. [11], the EVs for each trait must be derived independently from other breeding goal traits. In order to avoid the double counting of effects, the simulated economic outcome from SimHerd was corrected using multiple regression analysis. The correlations between the traits can be understood as indirect pathways (referred to as mediator effects) from the trait of interest to the simulated outcome. Therefore, in the present study, the economic outcome was corrected for inter-relations between traits to avoid double counting when deriving EVs, as proposed by Østergaard et al. [11]. The mediator variable has to be modeled as correlating with the traits of interest in SimHerd, and also has to be a part of the breeding goal with an own EV [11]. For example, ketosis in dairy cattle causes substantial losses in milk yield even before any clinical symptoms in sick cows are visible [23]. However, the economic consequences of reduced milk yield due to the sickness of cows must not be accounted for in the EV of ketosis since milk yield is also a part of the breeding goal. In Table 2, traits used as mediator variables in the regression analyses of disease traits and dystocia are presented.

Milk yield was used as a mediator variable of all diseases. The EV of mastitis was corrected using milk yield and cow mortality as mediator variables. The EV of metritis was corrected using milk yield, ketosis, cow conception rate and cow insemination as mediator variables. Milk yield and cow conception rate were used as mediator variables in the regression analysis of ketosis. Milk fever was corrected using milk yield, metritis, mastitis and dystocia as mediator variables. In the regression analysis, lameness was corrected using milk yield, cow conception rate and cow mortality. Dystocia was corrected using stillbirth and cow mortality. The regression model can be described as follows [11,13,21]:

(1) $$NetReturn_{ijkl}=\mu +\beta_{a}\;x_{ij}+\sum_{k=1}^{n}\beta_{bk}\;m_{k}+\beta_{c\;}\;x\_diff_{ij}{}_{\;}+\varepsilon_{ijkl}$$

where *NetReturn_ijk_*_l_ is the average annual net return, resulting from the i-th simulated replicate (i = 1,…,1000) for the j-th simulated level (j = 1,2) of the trait *x_ij_. ß_a_* denotes the corresponding regression coefficient and, at the same time, represents the estimate of the EV of trait *x_ij_.* As *μ* denotes the fixed intercept, *ß_bk_* is the regression coefficient of the mediator variable *m_k_*, where each of the n mediator variables have to be considered in the regression. Since SimHerd provides stochastic simulation elements, *x_diff_ij_* is included with its regression coefficient *ß_c_* to account for independent random variation within the simulated risk level and *ε_ijkl_* is the random residual error. Table 2 provides information in which mediator variables *m_k_* were used in the regression analyses to correct regarded trait *x_ij_* [11].

### 2.4. Input Parameters for Model Calibration

The simulation model was calibrated for the LR, LROG and LBW breeds using breed-specific phenotypic data. Milk and reproductive performance data were collected from the productivity reports of the Lithuanian Controlled Cow Herds for 2016–2017, no. 81 [24], and are shown in Table 3 and Table 4. In these tables the following parameters were specified for each breed separately: fat and protein (%), energy corrected milk (ECM), calving interval estimated in days, age at 1st calving estimated in months, heat observation rate for cows and heifers, conception rate for cows and heifers and start of breeding, estimated in days.

Table 4 presents the parameters that were used in the calculations: the calving interval was the shortest in LR, and it was 12 and 9 days longer in LBW and LROG, respectively, compared with LR. The age at 1st calving (months) was the highest in LBW. The heat observation rate for cows was the highest in LR and LROG. The conception rate for cows was 5% lower in LR than in LBW and LROG. This parameter was calculated by the formula (1/no of insemination) × 100). The number of inseminations was 2.0 in LBW and 2.2 in LR. The conception rate for LROG cows was simulated using the SimHerd program.

The information about the diseases of the breeds is presented in Table 5. The following input parameters were specified: incidence rates of stillbirth, milk fever, dystocia, metritis, ketosis, mastitis and lameness. The levels of prices and costs used in the simulations are presented in Table 6. The prices and costs were the same for all the breeds except for LBW, where the prices for pregnant heifers, unpregnant heifers, bull calves and semen were higher.

## 3. Results

### 3.1. Herd Statistics: Income and Costs for Lithuanian Dairy Breeds

For the better understanding of the simulated economic outcome for each breed, all relevant income items and cost positions are given in Table 7.

The annual economic results were calculated by the income from milk, the slaughter of heifers, calves and heifer life and costs (feed for cows, feed for heifers, insemination for cows and heifers, veterinary and other costs). The annual economic results were calculated by deducting costs from the income. The analysis shows that the income from milk is one of the main sources of income throughout the year. In this analysis, the income from milk differs between LROG and LBW and also LROG breeds. The results showed that the largest income from milk was from LR and LBW, while the lowest from LROG. Feed for cows accounted for the largest share of expenditure in all the breeds. However, it should be noted that LROG showed the lowest cost per cow (LR + EUR 142, LBW + EUR 129). Table 7 indicates that the highest profit (EUR/cow) was found in a herd of LR (EUR 794), then followed LBW (EUR 756) and the lowest was that of LROG (EUR 474).

### 3.2. Economic Values

To demonstrate the importance of health traits, we provide reliable EV estimates (Table 8) for functional traits related with herd health. The EVs were expressed in Euro per marginal change in the unit of the trait and cow-year for the three cattle breeds in Lithuania.

#### 3.2.1. Economic Values of Production

The EVs of energy corrected milk (ECM) was 0.16 EUR/kg for LBW and LR, and EUR 0.21/kg for LROG. The higher EV for LROG is caused by a lower feed intake and a lower milk yield, compared with LR and LBW, as it was assumed that a larger proportion of concentrated feed was needed to ensure increased milk yield.

#### 3.2.2. Economic Values of Direct Health Traits

**Figure 1:**
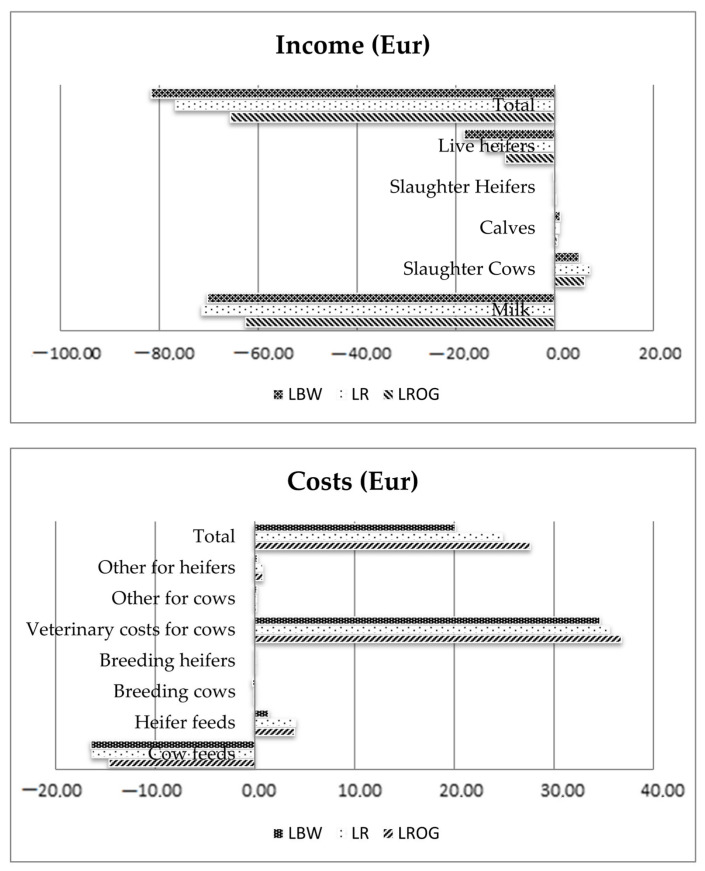
Economic incomes and costs (in Euro) caused by an increase in mastitis rate of 1 percentage point for the breeds LBW, LR and LROG.

The EVs of disease traits are expressed as economic consequences per cow-year due to an increase in mean disease incidence rate by one percent. If the EV is multiplied by 100, it expresses a total cost per case of the respective disease. The EVs of all health traits were highest for LBW, slightly lower for LR and lowest for LROG on the marginal scale. The highest EVs were found for mastitis in all three breeds. The results indicated the total costs of EUR 164 to 182 per case of mastitis, including direct costs, such as veterinary treatment, financial losses due to withdrawal of milk and additional labor costs. The present study revealed the total economic costs of EUR 107 to 127 per case of lameness. The EVs for direct health traits were found for ketosis (EUR 101 to EUR 130 per case), milk fever (EUR 114 to EUR 126 per case) and metritis (EUR 95 to EUR 100 per case). The EVs of direct health traits were affected by the differences in milk yield, for example, a lower milk yield performance of the LROG breed leads to lower economic values because of fewer economic losses associated with withdrawal milk. Due to the fact that mastitis was characterized by the highest values among economic health traits, a study was carried out to analyze in more detail the income and costs in relation to mastitis increase. The data presented in Figure 1 indicate the change per cow in the income from milk, carcass, calf and heifer selling and costs related to heifer and cow feeding, insemination and disease treatment by analyzing the differences of the two different scenarios in all the breeds.

The analysis of the income indicated that a negative economic return for milk, i.e., lost sales, was typical for all the breeds, but the highest was found for LR (EUR −71.36) and LBW (EUR −70.22), whereas the lowest was for LROG (EUR −62.47), and this was the results of the difference in milk yields. The highest income from slaughtered cows was found in LR and the lowest in LBW breeds. The losses for the unsold heifers were different, a low loss was found in LROG and the highest loss in LBW. All the income was added as the total income, and the highest and lowest income was estimated for LBW and LROG breeds. Cow treatment and feeding made economic costs in all the breeds. The highest costs caused by mastitis were estimated in LROG. The largest aspect is veterinary costs for cows. These results may have been influenced by the fact that the age of lactation for LROG was the highest [24].

#### 3.2.3. Economic values of Calving

**Figure 2:**
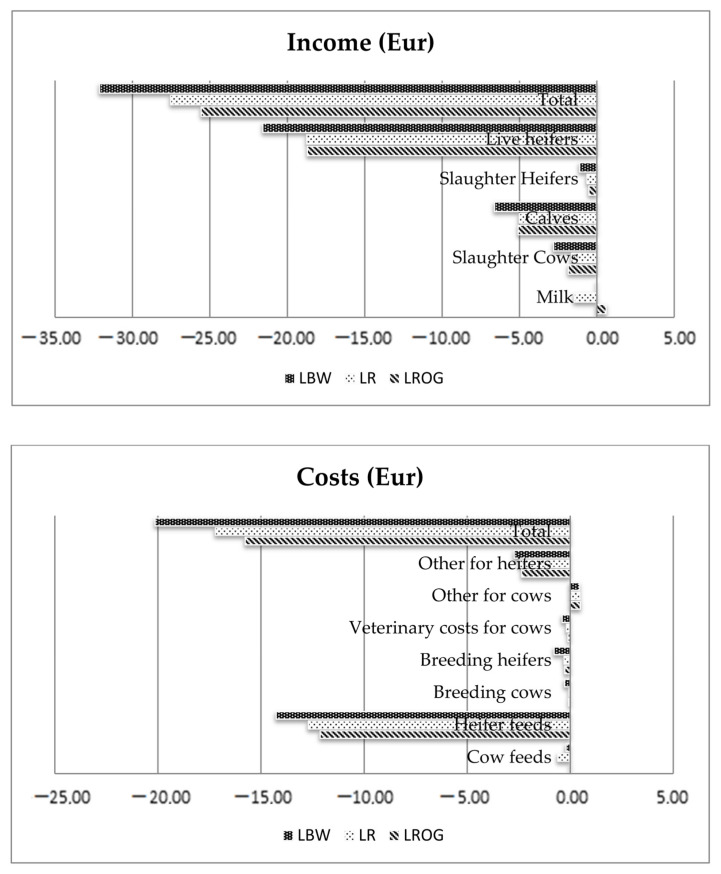
Economic income and costs (in Euro) caused by an increase in stillbirth rate of 1 percentage point for the breeds LBW, LR and LROG.

The highest economic values of dystocia were estimated for LROG (EUR −1.32), slightly lower for LBW (EUR −1.31) and LR (EUR −1.23). In order to avoid double counting, in the regression analysis dystocia was corrected by the mortality data of cows and calves. Dystocia involves direct expenses, such as veterinary treatment and additional labor force, and also economic impact due to herd information effect. LBW showed the highest stillbirth EV (in EUR per change in trait unit and cow-year), which was EUR −2.19, a slightly lower EV was found in LR (EUR −1.87) and LROG (EUR −1.76). Due to the fact that stillbirth was characterized by the highest values among calving traits, a study was carried out to analyze in more detail the income and costs in relation to stillbirth increase. The income and costs in relation to the increasing stillbirth rate are presented in Figure 2.

The analysis of the differences between “high“ and “low“ scenarios indicated that LBW demonstrated the highest lost income from live heifer, calf and slaughtered cow sales. The highest costs generated by the increased stillbirth rate were those of feeds for heifers.

#### 3.2.4. Economic values of Calf survival

**Figure 3:**
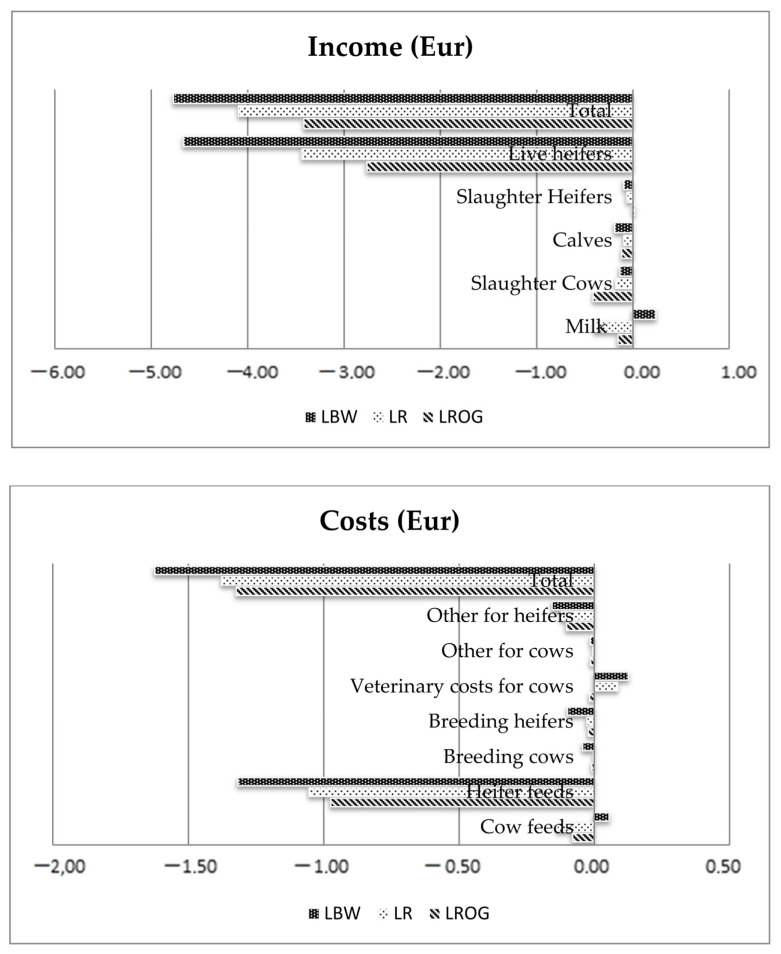
Economic income and costs (in Euro) caused by an increase in late calf mortality rate of 1 percentage point for the breeds LBW, LR and LROG.

The largest EV for early calf mortality were estimated in LBW (EUR −1.70), whereas lower EVs in LROG (EUR −1.30) and LR (EUR −1.14). The economic values for late calf mortality were similar (from EUR −3.49 to EUR −3.51) in LBW and LR, whereas that for LROG was EUR −2.63. The above values were influenced by carcass prices. The difference between high and low scenarios indicates that the income from live heifers is unequal, amounting to EUR −2.76, −3.43 and −4.67 for, respectively, LROG, LR and LBW. The highest late calf mortality expenses were caused by heifer feeding, costs (from EUR −0.97 to −1.32). The income and cost analyses of late calf mortality are presented in Figure 3.

#### 3.2.5. Economic Values of Cow Survival

**Figure 4:**
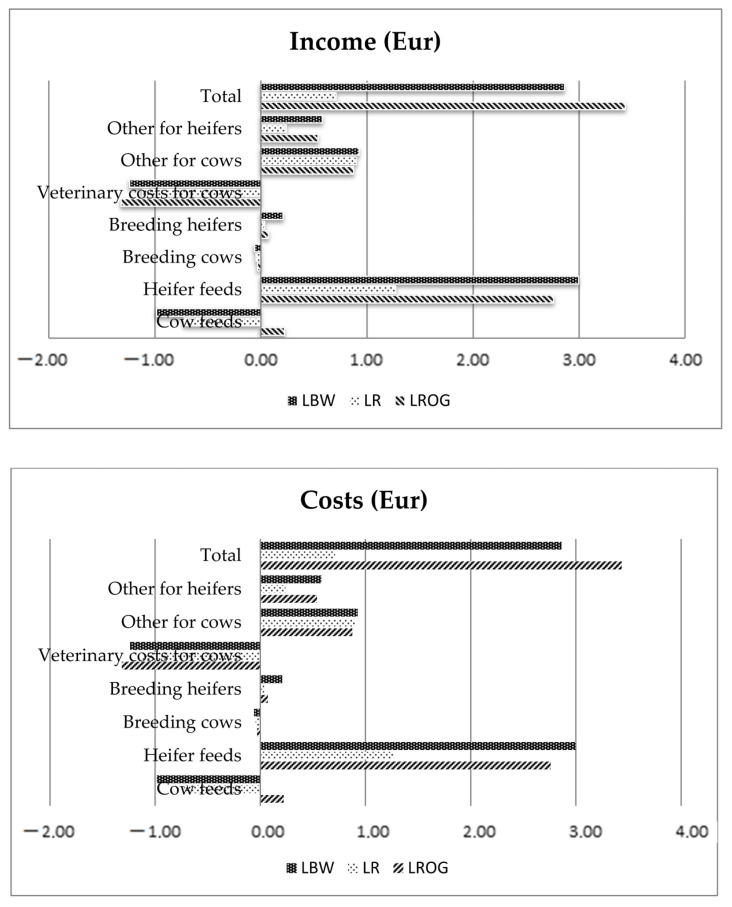
Economic income and costs (in Euro) caused by an increase in cow mortality rate of 1 percentage point for the breeds LBW, LR and LROG.

The highest economic loss between the breeds for cow mortality was estimated in LROG (EUR −918), LR (EUR −1144) and LBW (EUR −1077). The income and costs analyses of cow mortality are presented in Figure 4.

The analysis of the income indicated that a negative economic return for milk, slaughter cows and life heifers were estimated as the highest lost sales in LBW and LR breeds. All the income was added as the total income, and the highest and lowest income was estimated for LBW and LROG, respectively. The highest costs generated by the increased cow mortality rate were those of heifers feed and other costs for cows. All costs added, the highest were those of LROG and the lowest of the LR breed.

## 4. Discussion

To demonstrate the importance of health traits in herd management, reliable EVs estimates for functional traits related to herd health have been provided and expressed as the expected change in profit per cow-year. The information on cow health is becoming more important because of growing concerns about animal well-being and consumer demands for healthy and natural products [27]. Balancing fertility, udder health and metabolic diseases with high production is critical to herd management to maximize profits without compromising welfare [27]. Egger-Danner et al. [27] stated that many functional traits have negative genetic correlations with milk yield, and reductions in genetic merit for health and fitness have been observed. In our case, across the diseases, the highest EVs were found for mastitis and milk fever in LR and LROG, whereas mastitis and ketosis showed the highest EV in the LBW breed. The direct comparison of the EV between different countries is hardly possible, because of different economic assumptions and different production systems, but the tendencies of the EVs between different traits can be compared. According to Schmidtmann et al. [13], EVs of mastitis were highest in German dairy breeds with total economic costs per case of mastitis from EUR 257 to 271. Higher costs are primarily determined by higher treatment costs. A lower economic value of clinical mastitis (EUR 70.65 per case per cow and year) was estimated in a study with Pinzgau cattle [28]. Our results are, however, in agreement with results from other investigations, where mastitis is considered one of the most prevalent and costly diseases in dairy herds [29,30]. The second most pressing issue within the dairy industry is lameness; it has severe economic implications by causing a serious impact on animal welfare [31]. According to Ozsvari [32], the magnitude of loss resulting from lameness in dairy units is very similar in different countries, mostly varying between EUR 100–300 per case. According to our data, lameness in dairy cattle populations takes the third place across the estimated diseases, while in German breeds, mastitis and lameness had the highest EV [13]. According to Enting et al. [16], on Dutch dairy farms, claw lameness ranked third after mastitis and fertility problems. Among all the diseases, the second highest EVs were found to be ketosis in LBW and milk fever in both LR populations, and both diseases are known as disorders related to metabolic health in dairy cattle.

The estimated EVs for milk production traits in 305 days were higher for LROG and as they were affected by lower feeding costs for low production breeds. Similar results were obtained from Polish Holstein (PH) and Polish Red (PR) cattle [21]. Calving performance is considered as an important functional trait in dairy cattle [33]. Calving difficulties may cause injuries for both cows and calves and there is a higher possibility for increased stillbirth, lower milk production and the impaired health of cows. According to Mahnani et al. [34], stillbirth is an economically important trait on dairy farms and the knowledge of the consequences of and the economic losses associated with this trait can help the producer when making management decisions. LBW showed the highest stillbirth EV, which amounted to EUR 2.19 per change in trait unit and cow-year. These results are presumably influenced by 33% higher selling price for LBW calves in comparison with LROG and LR. Dystocia involves direct expenses, such as veterinary treatment and additional labor force, and also economic impact due to herd information effects. Kosińska–Selbi et al. [21] estimated the EVs of calving traits for Polish dairy cattle breeds using a bio-economic model. The economic value of stillbirth was observed for the Polish Holstein (EUR −1.53) and Polish Red (EUR −1.67) breeds, and that of dystocia for Polish Holstein (EUR −0.94) and Polish Red (EUR −1.26). The results of early calf mortality in Lithuania were influenced by higher selling prices for LBW nonpregnant and young heifers. The price for LBW of nonpregnant heifers was EUR 250 higher than that for Lithuanian Red Cattle, which could be explained by the fact that in Lithuania the population of Lithuanian Black-and-White cattle is dominated by 50–87.5% Holstein blood cows. In a dairy herd, heifers are very important as future replacement for milking cows and should be reared in an optimal way to maximize health, welfare and future prospective of milking cows [35].

Cow mortality in the bio-economic model is described as the on-farm death and as the total loss of a cow from the herd. The costs, such as production losses and treatment costs that preceded the death of the cow, are not considered. The economic effect involved in the economic value of cow mortality means profit loss as the animals are not sold for slaughter, also heifer replacement and carcass extermination expenses. The analysis of Polish Red cattle breeds indicated that the EV for cow mortality was from EUR 1208 to EUR 1239 [21].

In Lithuania, direct health traits have not been included in the national genetic evaluation yet, due to the lack of recorded data, but they are extremely important and help to improve herd profitability. In contrast to Germany, Norway, Denmark, Sweden and Finland, where direct health traits are officially introduced in the national genetic evaluation [28,36,37]. Therefore, all health trait characteristics should be recorded on farms and subsequently included in herd management programs.

## 5. Conclusions

The bio-economic simulation model SimHerd appeared to be a suitable tool for the EV derivation of the functional traits of Lithuanian dairy breeds. Overall, the results of this study allow to set up new breeding goals for all breeds and at the same time demonstrate the importance of health traits in herd management. The economic evaluation of the functional traits analyzed in this study indicated high economic importance of the functional traits in LR, LROG and LBW breeds. This study concludes that the development of joint breeding goals for health traits is very important for the best economic results. The balanced breeding goal reflects not only production, fertility or calving but also derives health trait benefits and positive economic value. The calculation of the EV, including animal health status, would allow the increase in the interest of dairy herd management by implementing on-farm methods for health status and dairy cow production analyses and development schemes that would decrease the number of disease cases and limit the use of drugs.

## Figures and Tables

**Table 1:** Schematic diagram showing stages of the SimHerd model.

1.	2.	3.
Lithuanian Black-and-White cattle open population (LBW)	Lithuanian Red cattle open population (LR)	Lithuanian Red cattle old genotype (LROG)
Simherd—mechanistic, dynamic and stochastic dairy herd model Monte Carlo model prediction of the production and states of the herd time		
2 STEPS:		
1. INPUT DATA:		2. OUTPUT DATA:
Parameter values for relations in the model: Breed calibration with the online version of SimHerd (https://simherd.com/en/, accessed on 25 January 2022): the simulation model was calibrated for the breeds, taking into account breed-specific phenotypic data from productivity Annual Reports of Lithuanian Breeds. The average herd size—200 cows. Simulated 40 years. First 10 years were deleted in order to diminish the effect of the actual state of the herd in the first simulation time-step. Replicated in 1000 simulations runs. Cows and heifers are described dynamically in weekly steps.		Technical annual results: Simulated scenarios were studied by applying a set of assumed Lithuanian prices and costs for the corresponding technical results. Scenarios were simulated to represent dairy herds with “low“ and “high“ levels of the trait. Performing multiple regression analysis to avoid double counting. The economic profit (EUR/year) of the simulated dairy herds is estimated mechanically as the difference between the total revenues and total costs [11,18].

**Table 2:** Mediator variables used in the regression analyses for the regarded traits.

Trait	Traits Used as Mediator Variables
Mastitis	Milk yield Cow mortality
Metritis	Milk yield Ketosis Cow conception rate Cow insemination rate
Ketosis	Milk yield Cow conception rate
Milk fever	Milk yield Metritis Mastitis Dystocia
Lameness	Milk yield Cow conception rate Cow mortality
Dystocia	Stillbirth Cow mortality

**Table 3:** Mean phenotypes for 305-d ECM, fat and protein (kg) for the LBW, LR and LROG.

Item	LBW	LR	LROG
Fat%	4.31	4.43	4.56
Protein %	3.35	3.50	3.48
1st lactation. kg ECM *	6741	6907	4916
2nd lactation. kg ECM *	7648	7596	5553
3rd lactation. kg ECM *	7526	7469	5949

* ECM = energy-corrected milk.

**Table 4:** Mean values of reproduction traits assumed for LBW, LR and LROG.

Trait	LBW	LR	LROG
Calving interval [days]	424	412	421
Age at 1st calving [months]	27.2	25.8	24.6
Heat observation rate cows [%]	43.16	45.16	45.24
Heat observation rate heifers [%]	59.91	55.08	55.56
Conception rate cows [%]	50 *	45 *	50 **
Conception rate heifers [%]	62.5 *	58.82 *	62.5
Start breeding [days]	44.27	46.67	45.62

* Conception rate was calculated by the formula: (1/no of insemination) × 100. ** Simulated by SimHerd.

**Table 5:** Number of treatments (per 100 cow-years) for the respective diseases in the breeds LBW, LR and LROG.

Disease	Breed		
	LBW	LR	LROG
Stillbirth *	6.5	5.2	-
Milk fever **	3.5	3.5	3.5
Dystocia **	1.3	1.3	1.3
Metritis **	8.0	7.0	7.0
Ketosis **	4.4	4.4	4.4
Mastitis **	26	26	26
Lameness **	19	19	19

* Stillbirth–Genetics evaluation model of calf mortality and general calving, creating an index of features, 2015 [25]. ** Based on the 2018 Annual Report of Nordic Cattle Genetic Evaluation [26].

**Table 6:** Levels of prices and costs (in Euro) used for simulations.

Year 2019	LBW	LR	LROG
Price kg ECM delivered to the dairy ^1^	EUR 0.29	EUR 0.29	EUR 0.29
Price per kg live weight for slaughter cows ^2^	EUR 0.94	EUR 0.94	EUR 0.94
Price for a dead cow for fallen stock company ^3^	EUR 33	EUR 33	EUR 33
Price to dispose of a dead heifer ^3^	EUR 21	EUR 21	EUR 21
Price to dispose of a dead calf ^3^	EUR 9	EUR 9	EUR 9
Price of pregnant heifer ^6^	EUR 1300	EUR 1050	EUR 1050
Price of unpregnant heifer ^6^	EUR 800	EUR 550	EUR 550
Price of bull calves ^6^	EUR 200	EUR 150	EUR 150
Price per kg milk powder ^1^	EUR 2.02	EUR 2.02	EUR 2.02
Price per SFU of concentrates for heifers ^4^	EUR 0.23	EUR 0.23	EUR 0.23
Price per SFU of roughages for heifer ^4^	EUR 0.10	EUR 0.10	EUR 0.10
Treatment cost for a case of clinical Mastitis ^5^	EUR 89	EUR 89	EUR 89
Treatment costs for a case of Milk Fever ^5^	EUR 54	EUR 54	EUR 54
Treatment costs for a case of Dystocia ^5^	EUR 69	EUR 69	EUR 69
Treatment costs for a case of Metritis ^5^	EUR 72	EUR 72	EUR 72
Treatment costs for a case of Ketosis ^5^	EUR 70	EUR 70	EUR 70
Cost for semen ^6^	EUR 20	EUR 7	EUR 7

^1^ “Agro market” 2019, 2020 Lithuanian agricultural and food market information system; ^2^ Survey of farmers; ^3^ Rietavo Veterinary Sanitation (VAT covered 100%); ^4^ list of normative prices for biological assets and agricultural products in 2019; ^5^ LUHS Institute of Animal Science; ^6^ Private AI Centers.

**Table 7:** Simulated annual economic results (EUR/cow) in a herd of LBW, LR and LROG.

	Lithuanian Dairy Cattle Breeds		
	LBW	LR	LROG
Income			
Milk	2110	2148	1675
Slaughter	143	136	140
Calves	100	76	77
Slaughter of heifers	16	12	11
Heifers Live	63	75	73
Total income	2432	2447	1976
Costs			
Feed for cows	1027	1040	898
Feed for heifers	247	238	229
Insemination costs cows	35	13	12
Insemination costs heifers	13	5	5
Veterinary costs for cows	105	109	111
Other costs for cows	202	202	202
Other costs for heifers	47	46	45
Variables costs	1676	1653	1502
Total contribution margin	756	794	474

**Table 8:** Marginal economic values (in Euro per change in trait unit and cow-year).

		Marginal EV			
Trait Complex	Trait	Unit	LBW	LR	LROG
Production	ECM	kg	0.16	0.16	0.21
Direct health	Mastitis	% point	−1.82	−1.73	−1.64
	Lameness	% point	−1.27	−1.22	−1.07
	Ketosis	% point	−1.30	−1.13	−1.01
	Milk fever	% point	−1.26	−1.26	−1.14
	Metritis	% point	−1.00	−0.95	−0.98
Calving	Dystocia	% point	−1.31	−1.23	−1.32
	Stillbirth	% point	−2.19	−1.87	−1.76
Calf survival	Early calf mortality	% point	−1.70	−1.14	−1.30
	Late calf mortality	% point	−3.51	−3.49	−2.63
Cow survival	Cow mortality	% point	−10.77	−11.44	−9.18
Fertility	CR heifers	% point	0.81	1.04	0.71
	CR cows	% point	3.82	1.95	1.96
	HO heifers	% point	0.37	0.59	0.51
	HO cows	% point	2.74	1.63	1.75

## Data Availability

Data are contained within this article.
